# Antibacterial Potency of Medicinal Plants including *Artemisia annua* and *Oxalis corniculata* against Multi-Drug Resistance *E. coil*

**DOI:** 10.1155/2021/9981915

**Published:** 2021-06-01

**Authors:** Hassan Golbarg, Mohammad Javad Mehdipour Moghaddam

**Affiliations:** ^1^Department of Biology, University Campus 2, University of Guilan, Rasht, Iran; ^2^Department of Biology, Faculty of Science, University of Guilan, 5th Kilometer of Persian Gulf Highway, Rasht, Guilan Province, Iran 4199613776

## Abstract

Antibacterial activity of ethanolic and aqueous extracts of two medicinal plants including *Oxalis corniculata* (EtOc, AqOc) and *Artemisia annua* (EtAa, AqAa) as well as *A. annua* essential oil (EoAa) was investigated on multi-drug resistance (MDR) *E. coli*. Microdilution and agar well diffusion methods were used to determine the minimum inhibitory concentration (MIC), minimum bactericidal concentration (MBC) as well as the inhibition zone. The phytconstituents of these products were analyzed using Reverse-phase High- performance liquid chromatography (RP-HPLC) and gas chromatography-mass spectrometry (GC-mass). The order of bacteriostatic and bacteriocide rate of the products can be shown as follows: EoAa>AqOc>EtAa = AqAa>EtOc, but the bactericidal effect of *A. annua* extracts is higher than of *O. corniculata* based on the MIC/MBC ratio and the order is as follows: EoAa>EtAa = AqAa>EtOc>AqOc. The most potent product, i.e. EoAa with a 56.7% inhibition of all isolates, has the potential to substitute 13 used antibiotics including oxacillin, amoxicillin, ampicillin, amoxicillin-clavulanic acid, tetracycline, streptomycin, ciprofloxacin, ceftriaxone, cefazolin, cefuroxime, cefotaxime, ceftazidime and cefixime (*P <0.05*). Different terpenoids were detected and measured in EoAa and catechin flavonoids in extracts of both plants, quercetin in extracts of *O. corniculata* but it was only possible to detect chlorogenic acid polyphenol in AqAa. Due to the antibacterial activities of the studied products, more effective than some antibiotics and their edible consumption, these products can be suggested as an alternative to some antibiotics and food preservatives to fight against MDR *E. coli.*

## 1. Introduction

The emergence of resistance among Enterobacteriaceae as the most common human bacterial pathogen to a wide range of antibiotics, a great deal of burden is being forced on patients and healthcare systems [1]. The Enterobacteriaceae of pathogens accounts for 80% of Gram-negative bacterial isolates that cause a variety of human diseases, including urinary tract infections (UTIs). *Escherichia coli* is the most common cause of UTI, accounting for 85% and 50% of community-acquired and hospital-acquired UTIs, respectively [2]. *E. coli* bloodstream infection sources (BSIs) were found to have a urinary source in 34% of cases [3].

Also, *E. coli* is the most prevalent microorganism responsible for health-related infections in the United States, according to the National Healthcare Safety (NHSN) Network [1]. Antibiotic resistance is caused by misuse, overuse and under-use of antibiotics by humans and is thus hazardous and threatening to public health [4].

Antibiotic resistance has been reported around the world in *E. coli*, with more than 95% of cases treated with serious symptoms prior to any culture detection. The emergence of MDR *E. coli*, particularly in infections such as UTIs associated with treatment defects occurs after the antibiotics prescription without any culture and the widespread use of antibiotics in clinics [5, 6]. The multi-drug resistance (MDR) bacteria survive exposure to antibiotics, so common medication become ineffective and infection persist, increasing the spread of infection [7]. Therefore, MDR bacteria are considered as a challenge in medicine.

Studies on chemical compounds of natural origin, in particular plants, have been ongoing for a long time, so that certain plants extracts or other herbal products can be used as an alternative source for the fight against antibiotic-resistant bacteria.

Of the approximately 500,000 known plant species, only 1-10% is used as human and animal food. Herbs have long been used as drugs to combat infections. Since ancient times, plants have been used as a remedy against infection, and even today, plant products are used almost all over the world, so that about 50% of pharmaceutical products are of plant origin in the United States [4, 8].

The plant products are complex set of primary and secondary metabolites, and their biological and pharmacological function may be due to synergies between different chemical components. The chemical components and levels of active compounds in these products are very diverse due to the plant interaction with the environment and other organisms. Since the manufacturing process of herbal medicinal products is very complex, it is preferable to use pure chemical compounds of plant origin due to easy of prescription, study the mechanism of pharmacological action and monitoring their side effects [9, 10].

Different plant secondary metabolites such as flavonoids, terpenes, phenolic acids, alkaloids, carotenoids, tannins, coumarins as well as some primary metabolites, such as peptides, amino acids, and organic acids show antimicrobial activity. Flavonoids have more benefits among the secondary metabolites mentioned and are found in different vegetables, fruits and plants and exhibit various activities, including antioxidant, immunomodulatory, anti-inflammatory, anti-cancer, neuroprotective and antidiabetic effects [11–13]. Plants containing polyphenols, due to their antimicrobial and antioxidant activity, are most of the medicinal plants studied [14, 15].

In this study, the antibacterial effects of two plant species including *Artemisia annua* and *Oxalis corniculata* were investigated. The genus *Artemisia* (Family Asteraceae) belongs to a valuable aromatic and medicinal plant group consisting of approximately 300 species found in the northern hemisphere. In Iran, there are about 34 native *Artemisia* spp. [16, 17]*. A. annua* called Qinghaosu, Sweet Sagewort, Sweet Annie, Sweet Wormwood, Annual Wormwood, is an annual plant which is widely distributed in Europe, Asia and North America, originating in China [18–20]. This plant has been used to treat malaria since ancient times [19]. The anti-malarial property of this plant is due to the presence of artemisinin sesquiterpene, and this plant has the potential for commercial development because of this compound [21, 22]. Moreover, this plant has antimicrobial, anticancer, anti-inflammatory, antipyretic, antiparasitic properties due to the existence of secondary metabolites such as flavonoids, monoterpenoids, sesquiterpenoids, coumarins, and aliphatic and lipid compounds [23, 24].

*O. corniculata* is a member of the Oxalidaceae family, also known as creeping wood sorrel or procumbent yellow sorrel, or the sleeping beauty [25]. Of the Oxalidaceae family, there is only the *Oxalis* genus in Iran [26]. There are about 900 species of the *Oxalis* genus that are mainly distributes in central South America's temperate and subtropical regions, including some other parts of the world common in Africa, Philippines, China, warmer parts of India and Pakistan [27]. Only two species have been reported from Iran, including *O. corniculata* and *O. articulate* [26].

*O. corniculata* is a well-known herb that has an acidic taste because of the high oxalate level of its stems and leaves [28]. It is usually distributed in Africa and Asia's subtropical regions [25]. Phytochemical analysis of this plant showed the presence of a mixture of different fatty acids and revealed the presence of flavonoids, phenolic compounds, phytosterols, essential oils, glycosides, proteins and amino acids in its ethanolic and methanolic extracts. Flavones, calcium oxalate, citric acid and tartaric acid are found in its leaves [29].

The aim of this study was to investigate the antimicrobial potential of ethanolic and aqueous extracts of *A. annua* and *O. corniculata* as well as *O. corniculata* essential oil on MDR *E. coli* isolates and also to identify some antimicrobial compounds in their products.

## 2. Material and Methods

### 2.1. Bacterial Isolation

This study was conducted on 138 urine cultures were obtained from patients, both women and men, suspected to have an UTI. The freshly voided midstream urine samples were transferred to the laboratory to avoid contamination as quickly as possible on deliver. Urine specimen was inoculated on the Cystine Lactose Electrolyte-Deficient (CLED) agar using standard culture methods. The CLED plates were incubated for 24 h at 37°C. Urine samples that contain ≥10^5^ colony-forming per milli-liter (CFU/ml) are considered positive [30, 31]. After isolation of bacteria using culture, Gram staining and biochemical differential tests were used to identify *E. coli* [32]. For further analysis, the isolates were stored at -70°C in a Tryptic Soy Broth containing 15% glycerol.

### 2.2. Antimicrobial Susceptibility

Antimicrobial susceptibility testing was conducted by the Kirby-Bauer disc diffusion system on Mueller-Hinton agar. This test was carried out using the following antibiotic disks (oxoid) against *E coli* isolates: oxacillin (OXA) (5 *μ*g), ampicillin (AMP)(10 *μ*g), piperacillin (PIP)(100 *μ*g), amoxicillin (AMX)(25 *μ*g), amoxicillin-clavulanic acid (AMC)(20/10 *μ*g), fosfomycin (FOF)(200 *μ*g), tetracycline (TE)(30 *μ*g), ciprofloxacin (CIP)(5 *μ*g), streptomycin (STR)(10 *μ*g), chloramphenicol (CHL)(30 *μ*g), cephalothin (CEF)(30 *μ*g), cefazolin (CFZ)(30 *μ*g), cefixime (CFM)(5 *μ*g), cefuroxime (CXM)(30 *μ*g), cefotaxime (CTX)(30 *μ*g), ceftriaxone (CRO)(30 *μ*g), ceftazidime (CAZ)(30 *μ*g), meropenem (MEM) (10 *μ*g), imipenem (IMP)(10 *μ*g) and cefepime (FEP)(30 *μ*g) [33, 34].

### 2.3. Preparation of Extracts

The leaves of two plants, including *A. annua* (ASA52185) and *O. corniculata* (OXO28252), which were collected from the Lahijan and Rasht regions in Gilan Province in northern Iran, were used to extract and study the antimicrobial activities. The identification and confirmation of species was performed by Dr. Davod Bakhsi and then voucher specimens deposited in the botanical herbarium at the Faculty of Agricultural Sciences, University of Guilan, Iran (with identification code, as indicated in parentheses above). The leaf samples of the two plants were cleaned, dried in an oven at 45°C, ground into fine powder and weighed exactly, and then subjected to extraction with ethanol 70% and water solvents at room temperature using Soxhlet system [35].

In aqueous extraction, 1 g of the leaf powder is soaked in 10 ml of distilled water in conical flask and put at 90°C for 60 min. Finally, the resulting suspension is incubated as overnight on the shaker incubator at 37°C, 150 rpm. Aqueous extract obtained was separated from solid residues by filtration using Whatman No. 1 filter paper. For ethanolic extraction, 1 g of each grinned plant material was extracted by 15 ml ethanol 70% for 12 h with continuous vigorous shaking at 30 min intervals. Then, the mixtures were filtered through Whatman No. 1 filter paper. The filtrate was collected and condensed by the rotary evaporator at 80°C for ethanol removal [35].

The oil extraction was performed according to the European Pharmacopoeia. A 300 g of the leaf powder of *O. corniculata* was mixed with 1000 ml of distilled water in 1000 ml distillation flask and hydrodistilled using an apparatus of Clevenger type for 3 h. The distillation flask was put in the hot plate and, with the addition of boiling chips, allowed the sample to boil until the distillation was accomplished. The extract (distillate) was collected in receiver apparatus. The extracted fractions of leaves displayed two separate layers including the lower aqueous layer and an upper oily layer. The oil was separated from the aqueous layer using a separatory funnel by extracting it twice with chloroform. After filtration of extracted oil, the chloroform was removed using rotary evaporator at 35°C. The obtained oil was dried over anhydrous sodium sulphate, filtered, concentrated under vacuum and then stored at 4°C in refrigerator until used for antimicrobial analysis [36].

### 2.4. Antimicrobial Activity

To evaluate the antibacterial potency of extracts and essential oil, the agar well diffusion and broth micro-dilution susceptibility methods were used. Due to the long name of each plant product, the abbreviations of each of them are as follows: essential oil, ethanolic and aqueous extracts of *A. annua* = EoAa, EtAa and AqAa; ethanolic and aqueous extracts of *O. corniculata* = EtOc and AqOc. To perform the diffusion agar well diffusion test, plates containing Muller Hinton Agar (MHA) medium are prepared and then 10 mm diameter wells are made on the medium using a cork borer. After preparation of the MAH medium, a suspension with a 0.5 McFarland (1.5 × 10^8^ CFU/ml) density is prepared from the test isolate and spread on the surface of the medium using a swab moistened with bacterial suspension. About 50 *μ*l of each plant product (10^−1^ to 10^−7^) is inoculated into the well. All plates are incubated for 24 h at 37°C and then the diameters of the inhibition zones were measured [37].

In the broth micro-dilution susceptibility method, 96-well microplate was used to determine minimum inhibitory concentration (MIC) and the minimum bactericidal concentration (MBC) of plant products. A serial dilution of 10^−1^ to 10^−7^ was prepared from each product and 50 *μ*l of product, 50 *μ*l of MHA medium and 50 *μ*l of 0.5 McFarland suspension from the test isolate were added within each well. MIC and MBC of each product were determined after incubation for 24 h at 37°C. To determine MBC, the content of the well without growth was inoculated on the MHA plate and if the isolate did not grow on the plate, that concentration was considered as MBC [38]. The amount of product that inhibits or kills bacteria can be determined using MIC and MBC quantities. Antimicrobials are usually considered as bactericidal, if the MBC/MIC ratio is not more than 4 [39].

### 2.5. HPLC Analysis

The extracts were analyzed using Reversed- phase High- performance liquid chromatography (RP-HPLC) to exhibit and quantify of antimicrobial compounds including catechin, quercetin and chlorogenic acid. A 1 g of grind leaves was extracted with 6 ml of methanol and acetic acid extraction solvent (85 : 15, v/v). The obtained extract was filtered, concentrated and dried using a rotary evaporator. The dried extract was being dissolved in the mobile phase. Fifty *μ*l of prepared extract was injected in HPLC (Waters, 1525, Milford, USA) equipped with a UV-Visible detector (Waters Dual *λ* Absorbance 2487), C18 column: Waters Symmetry C18 5 *μ*m 5 × 150 mm (Waters, Dublin, Ireland), at 280 and 320 nm. The compounds were detected by comparing retention times and UV-DAD (280 and 320 nm) spectra with those for standard solutions. In order to measure phenolic acid in extracts, the integrated peak area was calculated, and the contents were estimated using the calibration curve by plotting the peak area against the concentration of the corresponding standard sample [40].

### 2.6. GC-MS Analysis

The *A. annua* essential oil components was quantified by Gas chromatography-mass spectrometry (GC-MS), an Agilent 6850 gas chromatograph coupled to an Agilent 7890A mass spectrometer. The components were separated on a HP-5MS UI capillary column (30 m, 0.25 mm, 0.25 *μ*m) including 5% phenyl polysiloxane as stationary phase. The oven temperature program was started at 50°C, retained for 3 min, then increased from 8°C min^−1^ to 250°C, and retained for 2 min. The Injector, interface and ion source temperatures were retained at 250, 250 and 220°C, respectively. The split injection (1 *μ*l) was carried out with a 1 : 50 split ratio and helium was applied as carrier gas with flow-rate of 1 ml/min. The spectrometers operated in the electron ionization (EI) mode and the scan mass range, the ionization energy and the scan rate were 3–500 m/z, 70 eV and 0.2 s per scan, respectively. The components of the essential oil were identified based on a comparison of their mass spectra with those of the NIST mass library [41].

### 2.7. Statistical Analysis

All measurements have been replicated three times, and data was reported as mean ± SD. The results were statistically evaluated using a one-way variance analysis (ANOVA) and the variations between the means were calculated using the multiple range tests of Duncan at *P ≤0.01*.

## 3. Results

### 3.1. E. Coli Isolates

**Figure 1:**
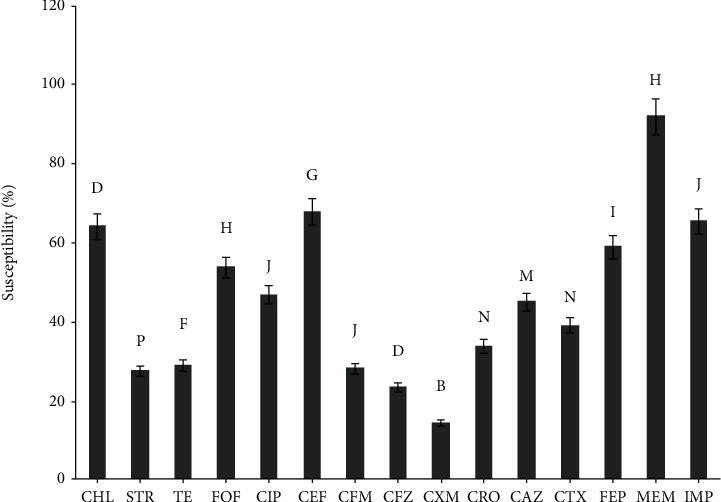
Susceptibility patterns of MDR *E. coli* isolates to different antibiotics. Values marked by different letters (a, b) are significantly different (*P* < 0.05).

*E. coli* were identified in 91 samples out of the 138 urine samples included in this study. Identification of *E. coli* based on observation of Gram-negative coccobacilli, *β*-hemolysis on blood agar, pink colonies on McConkey, greenish metallic sheen on EMB, indole production, positive MR reaction, nitrate reduction, enzymes production including catalase, Ornithine decarboxylase, *β*-glucosidase, CO_2_ production as well as glucose, lactose, manntiol and maltose fermentation. All *E. coli* isolates differ from each other, because they are distinct in their susceptibility pattern to the antibiotics used. The susceptibility pattern of the isolates to antibiotics used is shown in Figure 1. Since each isolate was resistant to at least three antibiotics, all isolates are referred to as MDR. Among the antibiotics used, meropenem and penicillins with the exception of piperacillin, were the most potent and weakest antibiotics against *E. coli* isolates, respectively. After meropenem (91.90% ±1.89), piperacillin (78.94% ±3.41) was the most effective antibiotic, followed by the three antibiotics including imipenem (65.34% ±1.79), cefepime (67.77% ±2.20), and chloramphenicol (64.07% ±3.15).

### 3.2. Antimicrobial Potency of Herbal Products

Preliminary antimicrobial screening test using agar well diffusion method was performed on 91 MDR *E. coli* isolates. Although a concentration range of 10^−7^-11.11 mg/ml was used to investigate the antimicrobial activity of the products, with the exception of one case of *O. corniculata* product and three cases of *A. annua* products, no activity of any of the products at concentrations below 0.0001 mg/ml was observed.

Some products had no effect on several isolates at the concentrations used. Some of them have an antimicrobial activity on one isolate, while the same isolate was not susceptible to another product from the same plant. According to the antimicrobial assay methods used, some products had a similar effect on different isolates and vice versa. Based on the results of agar well diffusion test shown in Table 1, *A. annua* extracts had a greater effect on more isolates than *O. corniculata* extracts. According to the data presented in Table 1, the most potent plant product used, EoAa, was capable of creating an inhibition zone (i.d) with a diameter of 20 ± 1.45 mm in 11.11 mg/ml concentration, although some extracts were able to make an i.d almost similar to EoAa. In the current study, the diameter range of i.d was from 4 ± 0.33 to 20 ± 1.45 mm. The mean i.d was higher in antibiotic- susceptible isolates than that of the studied herbal products.

**Figure 2:**
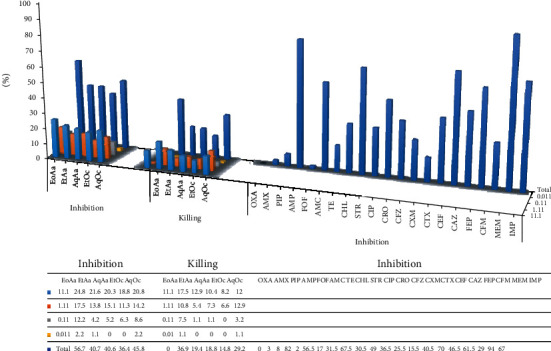
Comparison of the bacteriostatic and bacteriocide potency (%) of different concentrations (0.011, 0.11, 1.11 and 11.11 mg/ml) of two plant products with antibiotics against on MDR *E. coil*. EoAa, EtAa and AqAa = essential oil, ethanolic and aqueous extracts of *A. annua*; EtOc and AqOc = ethanolic and aqueous extracts of *O. corniculata*.

To accurately quantify the amounts of plant products as MIC and MBC that inhibit and kill isolates, the microdilution method was applied and their results were shown in Table 2 and Figure 2.

The AqAa and EtOc extracts had no inhibitory effect on any of the isolates at the lowest concentrations used, and the antimicrobial effects of EoAa and AqOc extract were similar. In general, EoAa and EtOc extract were the most potent and the weakest bacteriostatic products, respectively. The inhibitory activity was similar for both *A. annua* extracts, but the AqOc extract showed more bacteriostatic activity than the ethanolic type. The order of inhibition by the products can be shown as follows: EoAa>AqOc>EtAa = AqAa>EtOc. The product's bactericidal effect can be expressed both in terms of the MIC/MBC ratio (<4) and based on MBC alone. According to the latter case, the same inhibitory order is established for bactericidal activity, but the bactericidal effect of *A. annua* extracts is higher than that of *O. corniculata* extracts based on the MIC/MBC ratio (Table 3). Therefore, in comparing the two methods, the findings can vary, although the activity of EoAa in both methods is higher than that of all extracts from both plants.

The MIC and MBC values obtained from all products ranged from 10^−4^ to 11.11 mg/ml, and in MICs less than 10^−4^ mg/ml, none of the products had any activity on any of the isolates. In some cases, the MIC and MBC values for the products were the same, but in most cases the MBC values were higher than the MIC values. In *A. annua* products, there were three cases of MIC =10^−4^ mg/ml, including one case of EtAa extract and two cases of EoAa and also one case of MBC =10^−4^ mg/ml of EoAa. Also in *O. corniculata* extracts, two cases of MIC and two cases of MBC equal to 10^−4^ mg/ml by AqOc extract were displayed.

Of the 51 isolates affected by the activity of *A. annua* products, 12 isolates were susceptible to EoAa only, and 5 isolates were susceptible to EoAa and EtAa extract alone, and 2 isolates were susceptible to EoAa and AqAa extract alone. Exclusive inhibitory effects of the *A. annua* products were detected on 11 isolates at the concentrations used, i.e. the bactericidal effects were not detected on them. EtAa extract MIC was lower than AqAa extract on 10 isolates, while AqAa extract efficacy was higher than EtAa extract on 13 isolates. EtOc extract showed no activity on 8 isolates out of 39 isolates susceptible to antimicrobial activity of *O. corniculata* extracts and MIC of AqOc extract was also lower on 19 isolates than EtOc extract.

### 3.3. Herbal Products and Antibiotics

The bacteriostatic effects of various concentrations of plant products were compared with particular concentration of antibiotics, the findings of which are showed in Figure 2. The results are based on the number of isolates which are inhibited by different plant products concentrations. The most potent product, i.e. EoAa with a 56.7% inhibition of all isolates, seems to have the potential to substitute 13 used antibiotics including oxacillin, amoxicillin, ampicillin, amoxicillin-clavulanic acid, tetracycline, streptomycin, ciprofloxacin, ceftriaxone, cefazolin, cefuroxime, cefotaxime, ceftazidime and cefixime (*P <0.05*). Other products in this concentration have a bacteriostatic range of 36.4% to 45.8% (*P <0.05*).

### 3.4. Phytcompounds Analysis

**Figure 3:**
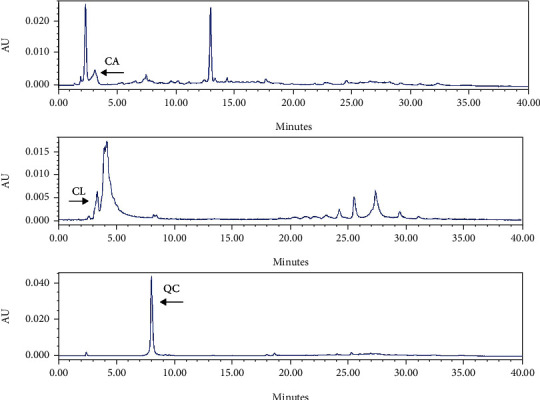
RP-HPLC chromatogram of the standards for catechin (CA, RT = 3.14 min), chlorogenic acid (CL, RT = 4.2 min) and quercetin (QC, RT = 8.00 min) at 280, 280 and 320 nm, respectively.

**Figure 4:**
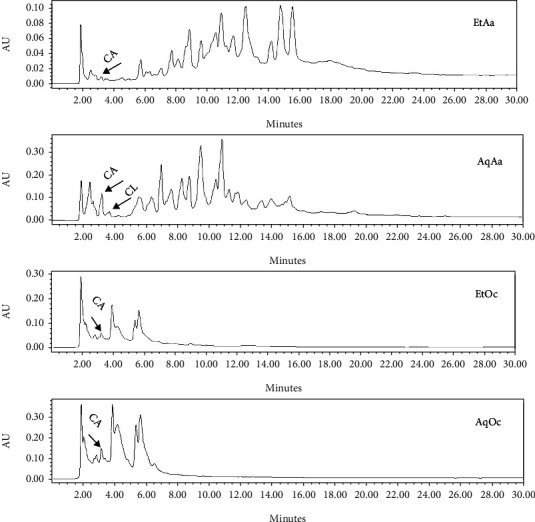
RP-HPLC chromatogram of catechin (CA) in EtAa (RT = 3.25 min), AqAa (RT = 3.31 min), EtOc (RT = 3.18 min) and AqOc (RT = 3.14 min) extracts and chlorogenic acid (CL) in AqAa (RT = 3.79) extract detected at 280 nm.

**Figure 5:**
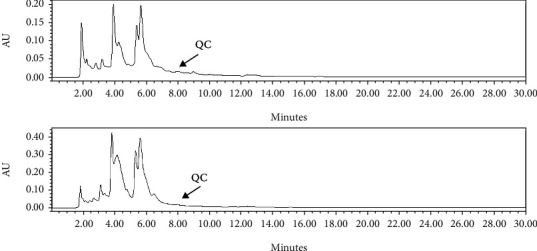
RP-HPLC chromatogram of quercetin in EtOc (RT = 7.90) and AqOc (RT = 7.97 min) extracts detected at 320 nm. This compound was not detectable in *A. annua* extracts.

RP-HPLC and GC-MS were used to analyze the presence of polyphenols, including catechins, quercetin and chlorogenic acid, in the extracts of both plants, as well as the constituents of essential oil, respectively. In order to identify polyphenols and quantify their amounts in the extracts, their standards have been used that the standard chromatogram of these compounds was demonstrated in Figure 3. Catechins were detected in extracts from both plants, but the amount was higher in aqueous extracts than in ethanolic extracts (Figure 4). The levels of catechin in each of the products, including EtAa, AqAa, EtOc and AqOc extracts were 0.166, 0.271, 0.195 and 0.348 g/100 g DW, respectively. Quercetin was not detected in *A. annua* extracts and was higher in aqueous extract (12.609 g/100 g DW) than in EtOc extract (4.311 g/100 g DW) (Figure 5). Chlorogenic acid could not be detected in extracts of both plants, except for the aqueous extract of *A. annua* (0.684 g/100 g DW) (Figure 4).

Phytochemical analysis of EoAa using GC-MS identified 31 peaks belonging to 7 types of compounds, including monoterpenes, sesquiterpenes, diterpene, cycloalkanes, cycloalkenes, alkyne and aldehyde (Table 4). Interestingly, about 50% of the EoAa constituents were terpenes, which included monoterpenes (16.25%) (alpha-pinene, camphene, 1,8-cineole, terpineol, Z-beta, cis-sabinene hydrate, borneol, Myrtenol, trans-(+)-carveol, and verbenene) and sesquiterpenes (21.8%)(alpha-copaene, rans-caryophyllene, germacrene, beta-selinene, bicyclogermacrene, caryophyllene oxide and ledene). The main terpenes contained germacrene (8.83%), 1,8-cineole (5.98%) and alpha-pinene (4.35%).

## 4. Discussion

Despite significant progress in medicine, combating pathogenic microorganisms remains a major challenge due to their resistance to antibiotics. Aside from the emergence of antibiotic resistance, the side effects of antibiotics have prompted researchers to seek out new antimicrobials, particularly those derived from plants [42]. Although several studies, especially on *A. annua*, have been conducted due to their anti-malarial properties, the antimicrobial activity of *A. annua* and *O. corniculata* extracts was investigated in this study because of the importance and necessity of replacing antibiotics with plant products [21].

In the current study, two antimicrobial testing methods including agar well diffusion and microdilution, were used to assess antimicrobial activity. The results of the two antimicrobial tests were almost consistent. For several reasons, well diffusion method has limitations and is only used for initial screening. First, since essential oils and their constituents are hydrophobic, they do not disperse evenly in the agar medium [43]. Second, because the exact quantity of extract diffused into the agar medium is unknown, the well diffusion and disk diffusion methods provide qualitative rather than quantitative data [40].

The antimicrobial activity of the essential oil was higher than that of the extracts among the *A. annua* products. The explanation for this may be due to a greater variety of antibacterial compounds, the antimicrobial efficiency of essential oil materials, the type of plant cultivar, or the solvents used in the essential oil extraction, preparation, and so on [44]. The essential oil of this plant was the subject of the majority of antimicrobial studies performed on it [21–24]. The extracts of this plant had almost similar bacteriostatic and bacteriocide effects. This contradicted the findings of other research. Donato et al. studied the antimicrobial activity of *A. annua* essential oil and its major components against seven foodborne pathogens. The i.d of 1.27 ± 0.31 mm and MIC =17.6 mg/ml was obtained of these products against pathogens such as *E. coli* O157 [38]. The analysis of their essential oils revealed 27 compounds, of which monoterpenoids constituted 1.4% and cisquiterpenes constituted 91% of the essential oil, whereas in this study, monoterpenoids and cisquiterpenes constituted 16.25% and 21.8% of the 31 compounds found in the essential oils, respectively. However, some essential oil constituents were found to be identical in both studies.

In another report, Prakash et al. found that a methanolic extract of *A. annua* had higher antibacterial activity on Gram-positive and Gram-negative bacteria than other extracts, though this extract's antibacterial activity was not observed on Gram-negative bacteria including *E. coli* and *Salmonella typhi*, but it did show almost significant activity on *Pseudomonas aeruginosa* (MIC =2 mg/ml and i.d = 17 mm) [37]. In the Kim et al. survey, the resistance of periodontopathic bacteria to various extracts of *A. annua* varied, with only the aqueous extract having activity on *Aggregatibacter actinomycetemcomitans* (MIC = 14 mg/ml) and this bacterium being resistant to other organic extracts [45]. The antimicrobial activity of different *Artemisia* species against pathogenic bacteria and fungi was found to be dependent on the solvent concentration used for extraction in the Hrytsyk et al. report [46]. Appalasamy et al. found that bioactive compounds isolated from *A. annua*, such as artemisinin and a precursor, had a poor inhibitory effect on Gram-positive bacteria such as *Bacillus subtilis*, *Staphylococcus aureus*, and *Bacillus thuringiensis*, similar to streptomycin. As a result, using only one component of an essential oil might not be efficient, even though using, preparing, and applying each component is simpler and more appropriate than using essential oils or extracts. Their results show that MICs less than 0.09 mg/ml inhibit the growth of extract-susceptible microorganisms [47]. This MIC value was within the range of the MIC values obtained from research.

Rolta et al. examined the antimicrobial effects of methanolic and petroleum ether extracts of *A. annua* on drug-resistant bacteria and fungi alone and in combination with antibiotics. Bacterial strains are more sensitive to *Candida* strains than to the bacteriostatic activity of these extracts, according to the results of their experiment. These extracts had a synergistic effect when combined with antibacterial and antifungal antibiotics, lowering MICs by 4-264 times against bacterial (*S. aureus* and *E. coli*) and *Candida* strains and increasing inhibitory activity. Using the diffusion method, none of these extracts showed antimicrobial activity against *E. coli* (ATCC25922) or *S. aureus* (ATCC29213), but methanolic extracts with MIC = 0.125 mg/ml and ether extract with MIC = 0.65 mg/ml inhibited both bacteria. In essential oil, thirteen compounds were identified, none of which were comparable to the essential oil compounds detected in this study [48].

According to the findings of Mamatova et al. ethanolic and chloroform extracts of *Artemisia gmelinii* may inhibit the growth of bacterial and *Candida* strains at MIC = 1.5-20 mg/ml. The extracts had the greatest bacteriostatic effect on yeasts, Gram-positive bacteria, and Gram-negative bacteria, respectively [40].

Hameed et al. evaluated the antimicrobial activity of *A. annua* methanolic extracts on *E. coli* and *S. aureus*. Both isolates had an i.d range of 0.70 ± 0.10 to 50 ± 0.20 mm, with *E. coli* having an i.d of 3.17 ± 0.15 mm, which was less than the minimum i.d (40.33 mm) obtained in this study. Antibiotics such as cefuroxime, streptomycin, and rifampin had a lower inhibitory effect on *E. coli* than methanolic extract, even though the inhibitory effect of the extracts used in this study was lower than streptomycin and cefuroxime. Phytochemical analysis of essential oils identified 59 compounds that were almost entirely different from the compounds found in the current research [49].

In the current survey, the extracts were assayed for antibacterial flavonoid compounds including catechins, quercetin and chlorogenic acid. Catechin appears to be working through many different mechanisms. It disrupts cell membranes by binding to bilayer membranes and inhibiting or inactivating the synthesis of intracellular and extracellular enzymes [50], as well as respiratory bursts by generating reactive oxygen intermediates (ROIs) [51], which disturb membrane permeability. Catechin also disrupts cell wall synthesis by binding to peptidoglycans [52]. Several antibacterial mechanisms, including DNA gyrase inhibition [53], ATP hydrolysis inhibition [54], and metal complexation [55], have been proposed for quercetin. Chlorogenic acid, like catechin, damages cell membranes [56].

Using liquid chromatography/diode array detector-atmospheric pressure chemical ionization/mass spectrometry (LC/DAD-APCI/MS) at 335 nm, Lai et al. and Han et al. were able to detect more than 40 compounds in the methanolic extract of *A. annua,* including chlorogenic acid and quercetin, while only chlorogenic acid was detected in the aqueous extract in the current study [57, 58]. Ivanescu et al. qualitatively detected chlorogenic acid in the methanolic extract of the aerial portion of *A. annua* and, in contrast to our research, was able to identify and quantify quercetin at 2.456 mg/100 g dry herb and 3.33.6 mg/100 g dry herb in the methanolic extract alone and treated with hydrochloric acid, respectively [59]. In the Carvalhoa et al. analysis, catechins, quercetin, and chlorogenic acid were detected in the methanolic extract of *A. annua* leaves in amounts of 79.53 ± 0.123, 0.74 ± 0.004, and 0.76 ± 0.022 mg/g dry matter, respectively. Catechin levels were approximately 35 times higher than those measured in AqAa extract (the highest amount of catechin and the only product containing chlorogenic acid) of our sample, but chlorogenic acid levels were about 9 times lower [60].

In this research, among *O. corniculata* products, aqueous extract had a higher antimicrobial potency than ethanolic extract. Adnan Siddiqui et al. reported that different *O. corniculata* extracts had different effects on Gram-positive and Gram-negative bacteria. According to their results, the most active extracts were aqueous and methanol extracts, which were comparable to tetracycline inhibition activity. Phytochemical analysis of extracts with the highest inhibitory activity indicated the existence of antimicrobial compounds such as phenolic compounds, alkaloids, flavonoids, and others [61]. In another study conducted by the Mukherjee et al. the bacteriostatic activity of methanolic extract of *O. corniculata* leaf was observed against pathogenic bacteria such as *E. coli*, *Shigella* spp. and *S. aureus*, so that the i.d ranged from 12 ± 0 to 19 ± 0.5 mm and the highest was correlated with *E. coli*. The MIC range was 0.08 ± 0.00 to 0.13 ± 0.00 mg/ml, with the lowest 0.08 ± 0.00 mg/ml against *E. coli*. In comparing extracts and antibiotics against *E. coli*, the extract had a higher bacteriostatic rate than the antibiotics ampicillin, doxycycline, and streptomycin, but the MIC value was different from the diameter determination, and the amount of MIC of ampicillin and streptomycin is half that of the extract against *E. coli* [62].

In a study conducted by Manandhar et al. that evaluated methanolic extracts from various *Oxalis* species against pathogenic bacteria, as well as *Aspergillus* and *Rhizopus* spp., only *O. corniculata* extract was effective against *E. coli*, while other bacteria, such as *P. aeruginosa* and *S. aureus*, as well as fungi, were not susceptible to this extract. In their analysis, the results of agar well diffusion and microdilution tests were not consistent [63]. In a study by Das et al. the antibacterial activity of *O. corniculata* aqueous extract as biofabricated silver nanoparticles (AgNPs) against UTI-causing bacteria was many times higher than normal extract [64]. Rahman et al. found that methanolic extract had more bacteriostatic activity against Gram-positive and Gram-negative bacteria than ethanolic extract, but the bacteriostatic activity of both extracts was lower than that of the cephachlore antibiotic [65]. In a different experiment, the antibacterial activity of *O. corniculata* leaf methanolic extract was reported to be greater than that of other organic extracts, as well as erythromycin and nalidixic acid, against a variety of bacteria, resulting in the highest rate of inhibition against *S. aureus* [66]. In a study that was almost similar to the previous two, the more potent activity of *O. corniculata* methanolic extract was reported in most cases, while chloroform, petroleum ether, and benzene extracts had no effect on at least eight human pathogenic bacteria. Interestingly, the effect of ethanolic extract on *E. coli* was greater than that of methanolic extract alone, and the sensitivity of bacteria to two antibiotics was higher than that of both extracts when compared to gentamicin and streptomacin. According to phytochemical analysis, the contents of compounds such as phenolic compounds and flavonoids are higher in methanolic and ethanolic extracts [28].

In analysis of phytochemical components and antibacterial potential of different parts of *O. corniculata* against *P. aeruginosa* and *Rhodococcus fascians* by Kaur et al., it was found that the leaves had more flavonoid and phenolic contents, and the seeds and stems had less phenolic and flavonoid contents, respectively. Leaves and seeds had the highest and lowest antibacterial activity, respectively [67]. Phytochemical assay of *O. corniculata* leaves grown in moist, marshy and dry areas revealed that 13 phenolic compounds, including quercetin (0.226 *μ*g/g), are more abundant in dry samples, followed by marshy and moist samples. However, the total flavonoid and phenolic content of the samples differed [68].

In most cases, the antimicrobial potency of the herbal products used in this research differed from that of other studies. Despite the fact that methanolic extract was not used in this study, results from other studies have shown that it is the most effective extract. Methanol is probably to be a suitable correlation with phenolic compounds, thus increasing its antimicrobial activities. The essential oils used in this analysis had different components and activities than those used in other studies, but some of the components were similar. However, the proportions of the components were different. This can be affected by method of extraction, distillation equipment, soil physicochemical parameters, plant age, plant cultivation techniques, density of plant population, harvest time, branch and leaf condition, relative humidity, the geographic environment, climate and managers [44].

## 5. Conclusion

The findings of the present study showed that the studied herbal products had almost significant antibacterial activity on MDR *E. coli* isolates, but in order to select a more appropriate and effective medication from these plants that can be used as an alternative to antibiotics, food preservative and as a candidate for treatment of UTIs, consideration should be given to methods and use of other solvents for extraction as well as further pharmaceutical analysis.

## Figures and Tables

**Table 1:** Antimicrobial potency of the herbal products against MDR *E. coli* isolates assayed by agar well diffusion method.

Isolates	EoAa	EtAa	Mean ± SD (mm)	EtOc	AqOc
			AqAa		
I1	8 ± 0.21	—	—	—	—
I2	—	—	—	—	—
I3	10 ± 0.12	7 ± 0.44	—	6 ± 0.24	11 ± 0.76
I4	9 ± 0.34	14 ± 1.60	8 ± 0.56	5 ± 0.33	—
I5	—	—	—	—	—
I6	—	—	—	—	—
I7	13 ± 0.46	—	—	—	—
I8	—	—	—	10 ± 0.46	14 ± 1.33
I9	11 ± 0.48	—	—	—	—
I10	16 ± 0.37	12 ± 1.46	10 ± 0.76	—	6 ± 0.76
I11	—	—	—	—	—
I12	7 ± 0.22	9 ± 0.33	—	—	—
I13	14 ± 0.46	11 ± 0.76	6 ± 0.48	11 ± 1.86	10 ± 0.76
I14	—	—	8 ± 0.27	10 ± 1.33	13 ± 0.87
I15	—	—	—	—	—
I16	—	—	—	—	6 ± 0.56
I17	6 ± 0.12	11 ± 0.76	—	5 ± 0.56	9 ± 1.76
I18	—	—	—	—	—
I19	7 ± 0.46	5 ± 0.22	7 ± 0.48	—	6 ± 1.15
I20	17 ± 2.48	12 ± 0.26	11 ± 0.35	—	—
I21	—	—	—	—	6 ± 0.76
I22	—	—	7 ± 0.33	—	—
I23	5 ± 0.27	—	—	—	—
I24	15 ± 0.64	6 ± 0.33	11 ± 0.64	—	—
I25	6 ± 0.33	5 ± 0.27	6 ± 0.45	—	7 ± 1.33
I26	7 ± 0.56	—	—	4 ± 0.33	4 ± 0.87
I27	—	—	—	—	—
I28	8 ± 0.36	6 ± 0.55	7 ± 0.12	5 ± 0.46	9 ± 1.66
I29	15 ± 1.76	10 ± 0.44	11 ± 0.55	—	—
I30	4 ± 0.36	4 ± 0.22		11 ± 1.54	13 ± 2.66
I31	—	—	—	—	—
I32	15 ± 0.46	20 ± 2.66	10 ± 0.74	14 ± 1.64	17 ± 2.65
I33	15 ± 0.28	13 ± 0.62	6 ± 0.33	—	—
I34	—	—	—	8 ± 0.67	12 ± 1.36
I35	6 ± 0.27	—	—	—	—
I36	7 ± 0.44	5 ± 0.21	7 ± 0.86	—	—
I37	—	—	—	9 ± 0.66	11 ± 1.66
I38	—	—	—	—	—
I39	8 ± 0.44	—	—	8 ± 0.64	11 ± 1.34
I40	6 ± 0.32	—	—	—	—
I41	—	—	—	10 ± 1.66	13 ± 2.64
I42	11 ± 0.27	7 ± 0.56	5 ± 0.24	—	—
I43	—	—	—	—	—
I44	6 ± 0.33	—	—	9 ± 0.76	9 ± 1.64
I45	—	—	—	—	—
I46	7 ± 0.44	5 ± 0.24	5 ± 0.78	—	6 ± 0.76
I47	17 ± 1.22	12 ± 0.76	7 ± 0.54	—	—
I48	7 ± 0.46	6 ± 0.33	8 ± 055	6 ± 0.54	8 ± 1.33
I49	—	—	—	—	—
I50	14 ± 0.46	14 ± 2.12	10 ± 0.62	—	—
I51	6 ± 0.46	—	—	9 ± 1.66	8 ± 0.64
I52	—	—	—	—	—
I53	7 ± 0.84	6 ± 0.33	—	8 ± 1.64	11 ± 2.33
I54	5 ± 0.24	7 ± 0.43	5 ± 0.36	—	6 ± 0.56
I55	15 ± 1.54	4 ± 0.16	11 ± 0.56	5 ± 0.33	8 ± 0.64
I56	—	—	—	—	—
I57	—	—	—	—	—
I58	6 ± 0.64	—	—	—	—
I59	16 ± 1.42	10 ± 0.22	12 ±1.89	7 ± 1.64	10 ± 2.64
I60	14 ± 0.76	5 ± 0.65	10 ± 1.20b	—	—
I61	10 ± 0.97	—	6 ± 0.76	9 ± 1.64	11 ± 1.66
I62	—	—	—	—	—
I63	—	—	—	—	—
I64	14 ± 1.60	10 ± 0.86	10 ± 1.40	—	—
I65	—	—	—	9 ± 1.76	9 ± 1.33
I66	—	—	—	—	—
I67	19 ± 2.60	5 ± 0.62	13 ± 1.60	—	6 ± 0.64
I68	—	—	—	—	—
I69	15 ± 2.30	7 ± 0.62	10 ± 1.60	—	—
I70	—	—	—	—	—
I71	—	—	—	—	—
I72	13 ± 1.85	6 ± 0.33	11 ± 1.62	9 ± 0.87	12 ± 1.87
I73	—	—	—	8 ± 1.64	7 ± 0.46
I74	—	—	—	—	—
I75	—	—	—	—	—
I76	—	—	—	11 ± 0.56	9 ± 1.64
I77	14 ± 2.60	—	10 ± 1.80	—	—
I78	—	—	—	9 ± 1.76	11 ± 2.64
I79	—	—	—	5 ± 0.12	8 ± 1.33
I80	9 ± 0.26	4 ± 0.26	10 ± 0.64	—	—
I81	10 ± 2.60	5 ± 0.86	10 ± 0.86	7 ± 1.66	9 ± 2.66
I82	—	—	—	—	—
I83	—	—	—	8 ± 0.64	7 ± 0.46
I84	—	—	—	—	—
I85	7 ± 0.22	—	5 ± 0.86	—	—
I86	—	—	—	11 ± 1.64	13 ± 2.76
I87	—	—	—	—	—
I88	15 ± 1.45	10 ± 1.24	14 ± 2.64	—	—
I89	6 ± 0.49	6 ± 1.33	8 ± 1.60	—	—
I90	—	—	—	—	—
I91	20 ± 2.45	11 ± 1.36	14 ± 2.45	5 ± 0.56	4 ± 0.24

All data presented was at a concentration of 11.11 mg/ml of the product. EoAa, EtAa and AqAa: essential oil, ethanolic and aqueous extracts of *A. annua*; EtOc and AqOc: ethanolic and aqueous extracts of *O. corniculata*.

**Table 2:** MIC and MBC values (mg/ml) of herbal products measured by microdilution method against MDR *E. coli* isolates.

Herbal products															
Isolates	EoAa			EtAa			AqAa			EtOc			AqOc		
	MIC	MBC	MBC/MIC	MIC	MBC	MBC/MIC	MIC	MBC	MIC/MBC	MIC	MBC	MBC/MIC	MIC	MBC	MBC/MIC
I1	0.1	>0.1	>1	—	—	—	—	—	—	—	—	—	—	—	—
I2	—	—	—	—	—	—	—	—	—	—	—	—	—	—	—
I3	0.1	>0.1	>1	0.1	>0.1	>1	—	—	—	0.1	0.1	1	0.01	0.1	10
I4	0.1	>0.1	>1	0.01	0.1	10	0.1	>0.1	>1	0.1	>0.1	>1	—	—	—
I5	—	—	—	—	—	—	—	—	—	—	—	—	—	—	—
I6	—	—	—		—	—		—	—	—	—	—	—	—	—
I7	0.01	0.1	10	—	—	—	—	—	—	—	—	—	—	—	—
I8	—	—	—	—	—	—	—	—	—	0.01	0.1	10	0.001	0.01	10
I9	0.01	0.01	1	—	—	—	—	—	—	—	—	—	—	—	—
I10	0.001	0.001	1	0.01	0.1	10	0.01	0.1	10	—	—	—	—	—	—
I11	—	—	—	—	—	—	—	—	—	—	—	—	—	—	—
I12	0.1	>0.1	>1	0.1	>0.1	>1	—	—	—	—	—	—	—	—	—
I13	0.001	0.001	1	0.01	0.1	10	0.1	>0.1	>1	0.01	0.1	10	0.01	0.1	10
I14	—	—	—	—	—	—	0.1	>0.1	>1	0.01	0.1	10	0.001	0.001	1
I15	—	—	—	—	—	—	—	—	—	—	—	—	—	—	—
I16	—	—	—	—	—	—	—	—	—	—	—	—	0.1	—	—
I17	0.1	>0.1	>1	0.01	0.1	10	—	—	—	0.1	>0.1	>1	0.01	—	—
I18	—	—	—	—	—	—	—	—	—	—	—	—	—	—	—
I19	0.1	>0.1	>1	0.1	>0.1	>1	0.1	>0.1	>1	—	—	—	0.1	—	—
I20	0.001	0.001	1	0.01	0.1	10	0.01	0.1	10	—	—	—	—	—	—
I21	—	—	—	—	—	—	—	—	—	—	—	—	0.1	—	—
I22	—	—	—	—	—	—	0.1	>0.1	>1	—	—	—	—	—	—
I23	0.1	>0.1	>1	—	—	—	—	—	—	—	—	—	—	—	—
I24	0.001	0.01	10	0.1	0.1	1	0.01	0.1	10	—	—	—	—	—	—
I25	0.1	>0.1	>1	0.1	>0.1	>1	0.1	>0.1	>1	—	—	—	0.1	—	—
I26	0.1	>0.1	>1	—	—	—	—	—	—	0.1	>0.1	>1	0.1	—	—
I27	—	—	—	—	—	—	—	—	—	—	—	—	—	—	—
I28	0.1	>0.1	>1	0.1	>0.1	>1	0.1	>0.1	>1	0.1	>0.1	>1	0.01	—	—
I29	0.001	0.01	10	0.01	0.1	10	0.01	0.01	1	—	—	—	—	—	—
I30	0.1	>0.1	>1	0.1	>0.1	>1	—	—	—	0.01	0.1	10	0.01	0.1	10
I31	—	—	—	—	—	—	—	—	—	—	—	—	—	—	—
I32	0.001	0.01	10	0.0001	0.001	10	0.01	0.01	1	0.001	0.01	10	0.0001	0.01	1000
I33	0.001	0.001	1	0.001	0.01	10	0.1	>0.1	>1	—	—	—	—	—	—
I34	—	—	—	—	—	—	—	—	—	0.01	0.01	1	0.001	0.01	10
I35	0.1	>0.1	>1	—	—	—	—	—	—	—	—	—	—	—	—
I36	0.1	>0.1	>1	0.1	>0.1	>1	0.1	>0.1	>1	—	—	—	—	—	—
I37	—	—	—	—	—	—	—	—	—	0.01	0.01	1	0.001	0.01	10
I38	—	—	—	—	—	—	—	—	—	—	—	—	—	—	—
I39	0.1	>0.1	>1	—	—	—	—	—	—	0.01	0.1	10	0.001	0.01	10
I40	0.1	0.1	1	—	—	—	—	—	—	—	—	—	—	—	—
I41	—	—	—	—	—	—	—	—	—	0.01	0.1	10	0.001	0.01	10
I42	0.01	0.1	10	0.1	>0.1	>1	0.1	>0.1	>1	—	—	—	—	—	—
I43	—	—	—	—	—	—	—	—	—	—	—	—	—	—	—
I44	0.1	>0.1	>1	—	—	—	—	—	—	0.01	0.1	10	0.01	0.1	10
I45	—	—	—	—	—	—	—	—	—	—	—	—	—	—	—
I46	0.1	>0.1	>1	0.1	>0.1	>1	0.1	>0.1	>1	—	—	—	0.1	>0.1	>1
I47	0.01	0.01	1	0.01	0.1	10	0.1	0.1	1	—	—	—	—	—	—
I48	0.1	0.1	1	0.1	>0.1	>1	0.1	>0.1	>1	0.1	0.1	1	0.01	0.1	10
I49	0.1	>0.1	>	—	—	—	—	—	—	—	—	—	—	—	—
I50	0.001	0.001	1	0.001	0.01	10	0.01	0.1	10	—	—	—	—	—	—
I51	0.1	>0.1	>1	—	—	—	—	—	—	0.01	0.1	10	0.01	0.01	1
I52	—	—	—	—	—	—	—	—	—	—	—	—	—	—	—
I53	0.1	>0.1	>1	0.1	>0.1	>1	—	—	—	0.01	0.01	1	0.001	0.01	10
I54	0.1	>0.1	>1	0.1	>0.1	>1	0.1	>0.1	>1	—	—	—	0.1	>0.1	>1
I55	0.001	0.01	10	0.1	0.1	1	0.01	0.1	10	0.1	—	—	0.01	>0.1	>10
I56	—	—	—	—	—	—	—	—	—	—	—	—	—	—	—
I57	—	—	—	—	—	—	—	—	—	—	—	—	—	—	—
I58	0.1	>0.1	>1	—	—	—	—	—	—	—	—	—	—	—	—
I59	0.001	0.001	10	0.01	>0.1	>10	0.01	>0.1	>10	0.1	0.1	1	0.01	0.1	10
I60	0.001	0.01	10	0.1	0.1	1	0.01	0.1	10	—	—	—	—	—	—
I61	0.01	0.1	10	—	—	—	0.1	>0.1	>1	0.01	0.1	10	0.01	0.01	1
I62	—	—	—	—	—	—	—	—	—	—	—	—	—	—	—
I63	—	—	—	—	—	—	—	—	—	—	—	—	—	—	—
I64	0.001	0.001	1	0.01	0.01	1	0.01	0.01	1	—	—	—	—	—	—
I65	—	—	—	—	—	—	—	—	—	0.01	0.1	10	0.01	0.1	10
I66	—	—	—	—	—	—	—	—	—	—	—	—	—	—	—
I67	0.0001	0.0001	1	0.01	0. 1	10	0.001	0.01	10	—	—	—	0.1	>0.1	>1
I68	—	—	—	—	—	—	—	—	—	—	—	—	—	—	—
I69	0.001	0.01	10	0.1	0.1	1	0.001	0.1	100	—	—	—	—	—	—
I70	—	—	—	—	—	—	—	—	—	—	—	—	—	—	—
I71	—	—	—	—	—	—	—	—	—	—	—	—	—	—	—
I72	0.01	0.1	10	0.1	>0.1	>1	0.01	0.1	10	0.01	0.1	10	0.001	0.001	1
I73	—	—	—	—	—	—	—	—	—	0.01	0.01	1	0.01	0.01	1
I74	—	—	—	—	—	—	—	—	—	—	—	—	—	—	—
I75	—	—	—	—	—	—	—	—	—	—	—	—	—	—	—
I76	—	—	—	—	—	—	—	—	—	0.001	0.01	10	0.001	0.01	10
I77	0.001	0.01	10	—	—	—	0.01	>0.1	>10	—	—	—	—	—	—
I78	—	—	—	—	—	—	—	—	—	0.001	0.1	100	0.001	0.1	10
I79	—	—	—	—	—	—	—	—	—	0.1	>0.1	>1	0.01	0.1	10
I80	0.01	0.01	1	0.1	>0.1	>1	0.01	>0.1	>10	—	—	—	—	—	—
I81	0.01	0.1	10	0.1	>0.1	>1	0.01	>0.1	>10	0.01	0.01	1	0.001	0.01	10
I82	—	—	—	—	—	—	—	—	—	—	—	—	—	—	—
I83	—	—	—	—	—	—	—	—	—	0.01	0.1	10	0.01	0.01	1
I84	—	—	—	—	—	—	—	—	—	—	—	—	—	—	—
I85	0.1	>0.1	>1	—	—	—	0.1	>0.1	>1	—	—	—	—	—	—
I86	—	—	—	—	—	—	—	—	—	0.001	0.01	10	0.0001	0.0001	1
I87	—	—	—	—	—	—	—	—	—	—	—	—	—	—	—
I88	0.001	0.01	10	0.01	0.01	1	0.001	0.01	10	—	—	—	—	—	—
I89	0.1	>0.1	>1	0.1	>0.1	>1	0.1	>0.1	>1	—	—	—	—	—	—
I90	—	—	—	—	—	—	—	—	—	—	—	—	—	—	—
I91	0.0001	0.001	10	0.01	0.1	10	0.001	0.001	1	0.1	>0.1	>1	0.1	>0.1	>1

**Table 3:** MBC/MIC ratio equal to 0, 1, >1, 10, >10, 100 and 1000 of various extracts on MDR *E. coli* isolates. If the bactericidal effect is expressed in terms of MBC/MIC ratio (<4), the bacterocide activity of *A. annua* products is weaker than that of *O. corniculata*.

Herbal products					
MBC/MIC ratio	EoAa	EtAa	AqAa	EtOc	AqOc
0	41	55	56	61	41
1	12	6	5	8	0
>1	23	16	16	6	4
10	15	13	9	15	17
>10	0	1	4	0	1
100	0	0	1	1	0
1000	0	0	0	0	1

**Table 4:** Phytoconstituents of EoAa detected by GC-MS analysis.

Peak no.	Compound	RT	Area (%)	Probability (%)
1	2-Hexenal	4.6	0.37	97
2	ALPHA.-PINENE	6.77	4.35	96
3	Camphene	7.23	0.74	98
4	Bicyclo[3.1.1]heptane, 6,6-dimethy l-2-methylene	8.12	0.57	97
5	1,8-cineole	10.06	5.98	98
6	1,5-Heptadien-4-one, 3,3,6-trimeth yl	11.04	5.9	83
7	Terpineol, Z-.beta	11.42	1.77	96
8	Cis-sabinene hydrate	12.53	1.13	97
9	2,3,3-Trimethyl-3-cyclopenteneace taldehyde	13.38	1.16	86
10	Bicyclo[2.2.1]heptan-2-one, 1,7,7- trimethyl	14.14	7.8	86
11	Bicyclo[2.2.1]heptan-2-one, 1,7,7- trimethyl	14.25	6.84	97
12	Methyl-2-methylene-	14.78	6.66	87
13	BORNEOL	15.13	4.77	97
14	3-Cyclohexen-1-ol, 4-methyl-1-(1-m ethylethyl)-	15.38	2.07	98
15	Bicyclo[3.1.1]hept-2-ene-2-carboxa	15.84	0.89	86
16	Myrtenol	15.9	1.36	93
17	TRANS-(+)-CARVEOL	16.68	0.52	98
18	Verbenene	19.27	1.02	80
19	Alpha.-Copaene	21.86	0.82	99
20	Trans-Caryophyllene	23.3	3.59	99
21	1,6,10-Dodecatriene, 7,11-dimethyl -3-methylene-,	24.42	1.48	98
22	Germacrene	25.31	8.83	98
23	Beta.-selinene	25.53	4.06	99
24	Bicyclogermacrene	25.65	0.69	97
25	Caryophyllene oxide	28.21	0.58	91
26	Ledene	29.53	1.64	92
27	Alpha.-Cubebene	29.8	1.59	90
28	7-Isopropenyl-4,4,10.Beta.-trimeth	30.18	0.46	90
29	7-Isopropenyl-4,4,10.Beta.-trimeth Yl-1,2,3,4,7,8,9,10-octahydronapht halene	32.1	1.46	90
30	2,6-Diethenyl-4-tert-butylphenol	32.35	0.86	78
31	Phytol	41.8	1.3	91
32			81.26	

## Data Availability

All data used during the study are available from the corresponding author by request.
